# Microorganisms’ Footprint in Neurodegenerative Diseases

**DOI:** 10.3389/fncel.2018.00466

**Published:** 2018-12-04

**Authors:** Mona Dehhaghi, Hamed Kazemi Shariat Panahi, Gilles J. Guillemin

**Affiliations:** ^1^Neuroinflammation Group, Faculty of Medicine and Health Sciences, Macquarie University, Sydney, NSW, Australia; ^2^Department of Microbial Biotechnology, School of Biology and Centre of Excellence in Phylogeny of Living Organisms, College of Science, University of Tehran, Tehran, Iran

**Keywords:** neurodegeneration disease, neuropathogenic microorganisms, gut microbiota, neuroinflammation, microbial infection, neurovirulence

## Abstract

Advancement of science has gifted the human a longer life; however, as neuron cells do not regenerate, the number of people with neurodegeneration disorders rises with population aging. Neurodegeneration diseases occur as a result of neuronal cells loss caused by environmental factors, genetic mutations, proteopathies and other cellular dysfunctions. The negative direct or indirect contributions of various microorganisms in onset or severity of some neurodegeneration disorders and interaction between human immune system and pathogenic microorganisms has been portrayed in this review article. This association may explain the early onset of neurodegeneration disorders in some individuals, which can be traced through detailed study of health background of these individuals for infection with any microbial disease with neuropathogenic microorganisms (bacteria, fungi, viruses). A better understanding and recognition of the relation between microorganisms and neurodegeneration disorders may help researchers in development of novel remedies to avoid, postpone, or make neurodegeneration disorders less severe.

## Background

Neurodegeneration is referred to neuronal cells loss, mostly with unknown reasons, that leads to various nervous system disorders. Neurodegeneration diseases such as Alzheimer’s disease (AD), amyotrophic lateral sclerosis (ALS), Huntington’s disease (HD), multiple sclerosis (MS) and Parkinson’s disease (PD) are considered as the significant health issues worldwide, contributing to high mortality rate in individuals which is sharply increased by aging. Among the neurodegeneration diseases, AD and PD have been known as the most prevalent neurological disorders based on World Health Organization (WHO) report (Hebert et al., 2013). A complex and outstanding relation between microorganisms and human have been developed from million years ago. Many human diseases have been caused by pathogenic bacteria, viruses, fungi and protozoa. A huge amount of data has proved that microorganisms derived infections are involved in chronic inflammation in human neurodegeneration diseases. Microorganisms can induce CNS dysfunction and neurodegeneration through various mechanisms. The host innate immune system is the first response of body against microorganisms’ infections that triggers during the first hours of infection. Accordingly, the activation of glial cells leads to production of cytokine and chemokine molecules that are associated with inflammatory response. Notably, it has been documented that neurons are also able to produce molecules related to immune system responses (Boulanger, 2009).

## Gut Microbiota

A complex, dynamic and large community of microorganisms termed “microbiome” or “microbiota” is present in human. A large number of these microbes present a symbiotic or commensal benefit to their host. Human gastrointestinal (GI) tract contains the largest community of microorganisms with about 10^14^ microbes from 1,000 different microbial species that possess about 4 × 10^6^ genes. More than 99% of GI microorganisms have been identified as anaerobic bacteria with other microbes including fungi, protozoa, viruses and archaea. This bacterial community comprises the largest microorganisms with density about 10^12^ per ml that is the highest microbial density in any ecosystem (Bhattacharjee and Lukiw, 2013). The most prevalent microorganisms in GI are *Firmicutes* (~51%), followed by *Bacteroidetes* (~48%). The other bacteria include *Cyanobacteria, Fusobacteria*, *Proteobacteria, Spirochaetes* and *Verrucomicrobia*. Recently, it was pointed out that microbiota can affect the health and diseases of human. Development of new methods such as new generations of sequencing and bioinformatics technologies has provided the opportunity to study the complex microorganisms-human interactions.

The communication of gut microbiota and the brain as well as the key role of this communication in the brain development and maintenance of the homeostasis have been demonstrated by modern physiology. The latter may be obtained through the down regulation of genes associated with immune system as well as regulation of blood-brain barrier integrity (Stilling et al., 2015). An increasing number of studies have emphasized the important role of microbiota in regulation of gut-brain axis, modulating of the brain function and providing a pathway to develop a bidirectional communication between the brain and gut (Foster et al., 2016). Recent studies reported that gut microbiota has a key role in the regulation of the neurotransmitters levels through adjustment of concentration of precursors or production of neurotransmitters. For example, *Bifidobacterium* and *Lactobacillus* species release the inhibitory neurotransmitter gamma-aminobutyric acid (GABA). Moreover, the production of norepinephrine by *Bacillus, Escherichia* and *Saccharomyces* species and serotonin by *Candida, Enterococcus and Streptococcus* has been reported (Lyte, 2014). Gut microbiota produces and releases other important metabolites such as short chain fatty acids (SCFAs). SCFAs including acetate, butyrate and propionate are the most significant metabolites affecting the CNS. The mediators of SCFAs actions are G-protein coupled receptors or histone deacetylases (Stilling et al., 2016).

*In vivo* studies have demonstrated that germ-free mice possess upregulation of some genes related to plasticity and specific metabolic pathways including steroid hormone metabolism, synaptic long-term potentiation and cyclic adenosine 5-phosphate-mediated signaling affecting particularly cerebellum and hippocampus regions. Moreover, germ-free mice showed significant elevation in 5-HT levels in hippocampus (Diaz Heijtz et al., 2011; Clarke et al., 2013). Interestingly, the concentration of brain derived neurotrophic factor (BDNF), a significant protein in learning, neuronal development, memory and mood regulation; reduced in germ-free mice. The evidence revealed that the expression of gene *Bdnf* decreased in the cortex and amygdale of germ-free mice (Neufeld et al., 2011; Clarke et al., 2013). Further studies have also implicated that neurogenesis can be controlled by gut microbiota. For example, hippocampal neurogenesis is influenced in germ-free mice due to modifications in the titres of substrates affecting hippocampal neurogenesis, including BDNF, corticosterone, pro-inflammatory cytokines and serotonin (Ogbonnaya et al., 2015). Some gut microbiota such as *Bacillus, Escherichia* and *Saccharomyces* species can produce norepinephrine. Moreover, serotonin can be synthesized by *Candida, Enterococcus* and* Streptococcus* (Lyte, 2014).

Prefrontal cortical myelination is also under regulation of microbiota. It is believed that germ-free mice exhibited an increase in volume of amygdale and also rendered dendritic hypertrophy in their basolateral amygdala (BLA). In addition, morphological changes in shape of pyramidal neurons of the BLA in germ-free mice have been observed (Luczynski et al., 2016). These observations emphasize the importance of the pre-weaning microbial colonization in gut.

Overall, gut microbiota may be recognized as a significant factor affecting the development of the CNS and its later function. These microorganisms are vital for anxiety-like behaviors, cognition, normal stress responsivity, sociability and maintenance of CNS homeostasis. Microbiota-derived CNS homeostasis is achieved through regulation of blood-brain barrier integrity and immune function. Neurogenesis, neurotoxicity, neurotransmitter level, neurotransmitter receptors, neurotropic signaling and synaptic systems can be influenced by gut microbiota.

The protective or negative role of gut microbiota in neurodegeneration and neurodevelopmental processes in some CNS diseases (Table 1) is discussed in detailed in following sections.

**Table 1:** The positive and negative effects of gut microbiota on central nervous system (CNS) function.

Biosynthesized metabolite	Impact on CNS	Function	Type	Gut microbiota example	Disorder
GABA	Inhibitory neurotransmitter	Inhibitory neurotransmitter reducing neuronal excitability throughout the nervous system	(−)	*BifidobacteriumLactobacillus*	AD, depression and synaptogenesis impairments
BMAA	Neurotoxicity	N-methyl-D-aspartate signaling dysfunction	(−)	*Cyanobacteria*	AD
Oxytocin	Positive-regulation of the neurotransmitters level	Improve social behavior and communication	(+)	*Lactobacillus reuteri*	Autism
-	-	Induction or suppression of autoimmune encephalomyelitis	Both	Different compositions	MS
Butyrate	Suppression of inflammation	Suppression of inflammation	(+)	*Faecalibacterium* *Coprococcus*	MS
Norepinephrine	Positive-regulation of the neurotrasmitters level	Improvement of alertness and arousal, and speeds reaction time	(+)	*Bacillus, Escherichia* and *Saccharomyces* species	Depression and hyperactivity disorder
Serotonin	Positive-regulation of the neurotrasmitters level	Regulation of anxiety, happiness and mood	(+)	*Candida, Enterococcus and Streptococcus*	Depression
SCFAs	Regulation of synaptic system	Induction of αSynpathology	(+)	-	NDs
PUFAs	Appropriate growth and function of nervous tissue	-	(+)	-	Autism
-	Reducing the removal of αSyn aggregates	Controlling cellular processes such as autophagy	(−)	-	Lewy body disease, multiple system atrophy and PD
Amyloids and LPS	Inducing inflammation	Production of pro-inflammatory cytokines Formation of A*β* fibrils Upregulation of steroid hormone metabolism, synaptic long-term potentiation, and cyclic adenosine 5-phosphate-mediated signaling.	(−)	*Escherichia coli*	AD
-	Adverse effects on growth and function of nervous tissue	Reduction of BDNF Significant elevation in 5-HT levels in hippocampus Immune system maturation Modified hippocampal neurogenesis Reduction of αSyn inclusion accumulation, motor deficits, and microglia activation	Both	Germ-free gut	Neurodegenerative and neurodevelopmental disorders

### Gut Microbiota Role in Parkinson’s Disease

Amyloidosis is referred to abnormal aggregation of proteins in neuronal cells and results in cellular functions disruption. It is supposed that the aggregation of insoluble proteins with altered conformation in tissues causes near to 50 human diseases. Amyloidosis contributes in some neurodegeneration diseases such as AD, HD and PD. Aggregation and accumulation of the protein α-synuclein (αSyn) in neurons, in particular dopaminergic neurons, is believed to be involved in synucleinopathies including multiple system atrophy, Lewy body disease and PD (Brettschneider et al., 2015; Prusiner et al., 2015). Based on Braak’s hypothesis (Del Tredici and Braak, 2008), aggregation of the protein αSyn appears early in enteric nervous system (ENS). Then, it is transmitted to the brain cells through vagus nerve. Pathophysiologic evidence has indicated that transferring of αSyn into the gut of healthy rodents can induce PD pathogenesis. Sampson et al. (2016) have studied the role of gut bacteria in pathophysiology of synucleinopathies and its connection with PD. The results suggested that gut microbiota is required to induce αSynpathology and motor deficits in mouse model. Interestingly, fecal microorganisms isolated from PD patients showed more destructive effects on motor function, compared to healthy controls. In germ-free and antibiotics treated mice, αSyn inclusion accumulation, motor deficits and microglia activation reduced, compared to animal models containing complex microbiota. A potential mechanism for αSyn-induced pathology involves the maturation and the activation of inflammatory pathways of microglia through SCFAs, produced by gut bacteria. The activation of microglia followed by the production of pro-inflammatory cytokines leads to neuronal cells death in PD models and other neurological disorders (Kannarkat et al., 2013). In addition, inflammation induces αSyn aggregation, activation of microglia and progression of neurodegeneration disorders. The gut bacteria have been shown to affect other cellular processes such as autophagy, a genetically process associated with PD, which may decrease the clearance of αSyn aggregates (Beilina and Cookson, 2016).

### Gut Microbiota Role in AD

There is a growing recognition of the significance of gut microbiota in the dissemination of beta amyloid (A*β*) in AD patients. Microorganisms usually excrete some complex products, such as amyloids and LPS, which are immunogenic for their host. Among them, microbial LPS can change gut microbiota homeostasis and induce inflammatory response as occurs in inflammatory bowel disease. Moreover, microbial amyloids are believed that involve in aggregation, formation of biofilms, invasion and colonization of pathogenic microorganisms. For example, *in vitro* studies have concluded that *Escherichia coli* was able to secrete an endotoxin, which involves in the formation of A*β* fibrils (Asti and Gioglio, 2014). Normally, amyloids and microbial LPS have a monomeric, soluble form; however, following aggregation and formation of insoluble structures, they may associate with oxidative stress and AD pathogenesis. The role of microbiota in amyloid formation becomes worthier of notice as gut epithelium and the blood-brain barrier permeability increase during aging. In addition, the age has direct relationship with the number of *Firmicutes* and *Bifidobacteria*. Nevertheless, the microbial composition of gut is also affected by diet, community and receiving different treatments. Recent studies have hypothesized that amyloids produced by gut bacteria can pass through the gut tract and accumulate in the brain (Zhao and Lukiw, 2015). This increases the amyloids induced oxidative stress and activates the nuclear factor-jB signaling leading to upregulation of pro-inflammatory microRNA-34a and A*β*42 peptide phagocytosis dysfunction by microglial cells. Moreover, microbial LPSs and amyloids aggravate the leakage of GI tract and induce production of pro-inflammatory cytokines, which leads to increase in AD severity.

As mentioned earlier, some gut bacteria such as *Bifidobacterium* and *Lactobacillus* species synthesize and release an inhibitory neurotransmitter, i.e., GABA. Modification in GABA signaling is associated with depression, AD and synaptogenesis impairments. One of the important residents of human GI tract is *Cyanobacteria* producing a neurotoxin called *β*-N-methylamino-L-alanine (BMAA). BMAA interferes with N-methyl-D-aspartate glutamate receptor and finally leads to N-methyl-D-aspartate signaling dysfunction in AD and other neurodegeneration diseases (Aziz et al., 2013).

### Gut Microbiota Role in Autism

Autism is a neurologic disorder associated with deficits in social behavior and communication. Basically, genetic has a key role in pathogenesis of autism; however, some documents revealed that more than 70% of autism subjects suffer from GI syndrome showing the role of gut microbiota in this disease. *In vivo* studies have indicated that germ-free mice have abnormal social behavior, compared to controls. Although behavioral improvement has been observed after microbial colonization, the behavior was not completely restored (Desbonnet et al., 2015). This may be because of the ability of gut microbiota in fermentation of SCFAs, which are essential for the production of polyunsaturated fatty acids (PUFAs). PUFAs are important for the brain development in respect to proper growth and function of nervous tissue. Low levels of PUFAs may be linked to neurodevelopmental disorders, for example autism, and associated with difficulty in behavioral and cognitive performance (El-Ansary and Al-Ayadhi, 2014).

Intriguingly, fecal microbiota examination has revealed the modifications in microbial community of intestine in autism patients. Based on microbial diversity analysis, it has been indicated that the ratio of *Bacteroidetes* to *Firmicutes* decreases whereas the population of *Lactobacillus* and *Desulfovibrio* species increases (Tomova et al., 2015).

Some gut microbiota such as the probiotic bacterium *Lactobacillus reuteri* can considerably elevate the oxytocin levels (Erdman and Poutahidis, 2014). Oxytocin is an essential peptide secreted by hypothalamus that is involved in social behavior and communication. Gene knock out of oxytocin receptor resulted in a significant behavioral deficiency in mice models proving the key role of gut microbiota in improvement of behavior.

### Gut Microbiota Role in MS

Recent studies have linked the composition of gut microbiota with MS severity. *In vivo* studies in mouse model of MS have revealed the ability of gut bacteria in induction of autoimmune encephalomyelitis after subjecting to myelin oligodendrocyte glycoprotein (Berer et al., 2011). In another study (Cantarel et al., 2015), the gut bacteria community of MS patients was compared to healthy individuals after treatment with vitamin D. Prior to vitamin D supplement, the abundance of some bacterial species such as *Faecalibacterium* was significantly lower in MS individuals than healthy subjects. After treatment of patients with vitamin D, the population of *Akkermansia*, *Coprococcus* and *Faecalibacterium* species increased in GI. This finding is in accordance with the fact that *Faecalibacterium* can contribute to the suppression of inflammation owing to its ability to produce butyrate. Similar to *Faecalibacterium*, *Coprococcus* may be also considered as an inflammation reducing bacterium (Cantarel et al., 2015). The examinations of microbiome community of relapsing-remitting MS patients and healthy subjects have also conducted by Chen et al. (2016). In the former individuals, the populations of genera *Blautia, Dorea, Haemophilus, Mycoplana* and *Pseudomonas* grew, whereas in the latter individuals, an increase in the populations of *Adlercreutzia, Parabacteroides* and* Prevotella* has been observed. In particular, the species richness increased in healthy individuals compared to MS patients. These studies highlight the gut microbial dysbiosis in MS patients, and consequently the significance of the gut microbiota in the etiology and pathogenesis of MS.

## Bacteria

### Mycobacterium leprae

**Figure 1:**
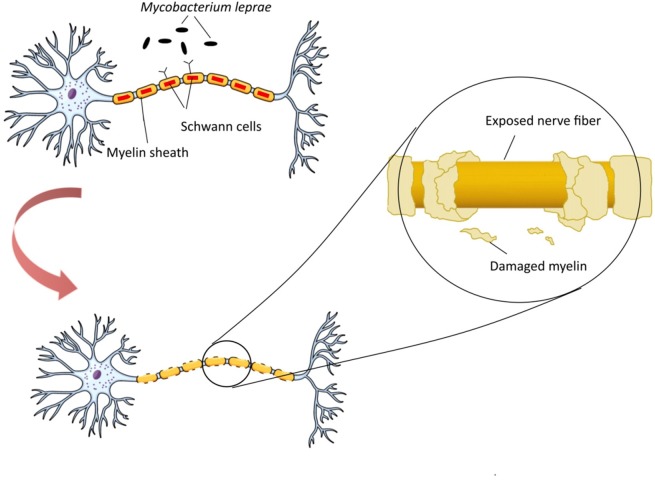
Neuropathogenesis of *Mycobacterium leprae. M. leprae* binds to myelin P zero (P0) on human peripheral nerve and colonize in Schwann cells. Upon this attachment and bacterial multiplication, infected Schwann cells undergo demyelination and start producing non-myelinated sheets, instead of myelinated sheets, to secure the intracellular niche for *M. leprae*. *M. leprae* reprograms Schwann cells into immature progenitor/stem cell-like entities to infect other tissues. Macrophages process and present *M. leprae* antigens to helper T-cells which cause their differentiation, and various inflammatory substances, such as γ-interferon (IFN-γ), are released. In the presence of IFN-γ, Schwann cells express major histocompatibility complex (MHC) class II on their surface and can present processed antigens of *M. leprae* to antigen-specific, inflammatory type-1 T-cells. Type-1 T-cells attack and lyse infected Schwann cells, which renders neurodegeneration disorders.

*Mycobacterium leprae* is a pathogen responsible for demyelination and nerve damage by targeting and manipulating the structure and function of Schwann cells in peripheral nerve. *M. leprae* can initially bind to a 28 kDa glycoprotein, myelin P zero (P0), a major human peripheral nerve protein which is specifically expressed in them (Vardhini et al., 2004). Binding of structurally similar molecules of *M. leprae* could disrupt the P0-P0 interactions leading to demyelination, a process commonly referred as contact dependent demyelination. Neuropathology of *M. leprae* is initiated with changing in milieu of the Schwann cells and activation of apoptotic pathways in cells, the feature of leprosy. This process triggers host autoimmune response against nerve cells antigens, followed by demyelination and cell death (Figure 1). Bioinformatics studies have revealed the similarity between *M. leprae* proteins and that of the human peripheral nerve, i.e., the binding site of this bacterium (Vardhini et al., 2004). More specifically, a 17–42-amino-acid-sequence in the secreted P60 family protein of *M. leprae* has similarity to the 51–56-amino-acid-sequence in the myelin P0 (within the immunoglobulin domain). This similarity suggests the role of P0 in protein-protein and protein-ligand interactions as well as the complication of autoimmunity in nerve system (Zhu et al., 2001). It is worth mentioning that immunoglobulin domain plays an important role in the interactions between proteins and ligands via homophilic adhesive properties (Brümmendorf and Lemmon, 2001).

### *Mycoplasma* Species

*Mycoplasma* species are the causative agent of respiratory diseases in human. Currently, the role of this bacterium in CNS disorders has gained considerable attention. *Mycoplasma* can be detected in samples using direct culture, PCR and Western blot. The latter two methods detect antibodies IgM and IgG against lipid-associated membrane proteins (LAMPs). The presence of *Mycoplasma* in bloodstream of ALS patients have been studied by Gil et al. (2014). According to this study, *Mycoplasma* species was detected in up to 46% of ALS patients and 9% of healthy individuals using culture or direct PCR methods. They also examined the sera for the detection of IgM- and IgG-specific to LAMPs of *Mycoplasma fermentans*. Forty six and 31% of patients with ALS respectively showed IgM and IgG against LAMPs of *M. fermentans*, compared to 7% for either of the antibodies in healthy individuals. Some blood samples from patient (46%) and healthy individuals (9%) also showed contamination with *Mycoplasma*. Intriguingly, *M. fermentans* was the identified *Mycoplasma* sp. in all the positive patients for *Mycoplasma* genus and in 50% of the positive healthy individuals for *Mycoplasma* (Gil et al., 2014). Similarly, Nicolson et al. (2002) examined blood samples of Gulf war veterans and civilians with ALS for the presence of *Mycoplasma* species. In the first group, all the individuals had blood mycoplasmal infections with *M. fermentans*, except one with *M. genitalium*. In contrast, about 79% (59% with *M. fermentans*) of the individuals in the second group were positive for at least one *Mycoplasma* sp., i.e., *M. fermentans*, *M. hominis, M. penetrans* and/or* M. pneumoniae* (Nicolson et al., 2002).

In overall, *Mycoplasma* can induce the activation of macrophages, monocytes and glial cells leading to production of inflammatory cytokines. The main mycoplasmal antigens are LAMPs which are one of the important target of humoral immune response. *Mycoplasma* may adversely contribute in the progression of ALS and/or its pathogenesis. Alternatively, patients with ALS may be extremely vulnerable to systemic infections with *Mycoplasma*.

### Chlamydia pneumoniae

*Chlamydia pneumoniae* is an intracellular bacterium that normally enters into the body through mucosa of the respiratory tract. It causes respiratory tract infections, i.e., acute bronchitis, chronic bronchitis and asthma, and community-acquired pneumonia in human. *C. pneumoniae* infection has been also reported in CNS disorders such as AD, meningoencephalitis and MS (Wunderink and Waterer, 2014). Access of the microorganism to the CNS thought to be via intravascular and olfactory routes. The presence of this pathogen in cerebrospinal fluid (CSF) of patients with either progressive MS or newly diagnosed relapsing-remitting MS was confirmed using direct culture, PCR and antibody detection for *C*. *pneumoniae* elementary body (EB) antigens. Approximately 86% of tested MS patients had enhanced levels of antibodies to *C. pneumoniae* EB antigens (Sriram et al., 1999).

*C. pneumonia* antigens were also present in the neocortex of AD and/or in association with senile plaques and neurofibrillary tangles (Choroszy-Król et al., 2014). In a separate study (Balin et al., 1998), nucleic acids from post-mortem brain samples of patients suffering from late-onset AD were prepared and compared to controls. PCR examinations for *C. pneumoniae* chromosomal DNA was positive for about 90% of AD patients whereas only 5% of control patients were PCR-positive. Additionally, *C. pneumonia* could densely culture from some AD brains whereas same culture studies of non-AD brain were negative for *C. pneumonia*. The regions of the brain including hippocampus, parietal cortex, prefrontal cortex and cerebellum were also examined by Electron- and immunoelectron-microscopic techniques. Interestingly, the chlamydial elementary and reticulate bodies were only identified from AD brain regions and not from non-AD brains (Balin et al., 1998). The presence of the microorganism in the brain was also confirmed by immunohistochemistry. *C. pneumoniae* antigens in astrocytes, macrophages and microglia of the hippocampus, parietal and prefrontal cortex and temporal cortices were detected only in AD patients, compared to controls (Balin et al., 1998). In a recent study (Paradowski et al., 2007), the presence of *C. pneumoniae* in CSF of 57 AD patients and its relationship with the levels of A*β*42 and tau protein was examined and compared to 47 controls. It was realized that the presence of chlamydial DNA in CSF of AD patients was significantly higher than control group; however, no effect on A*β*42 and tau protein levels in CSF could be linked to the activity of the microorganism (Paradowski et al., 2007). These studies clearly demonstrated the presence and viability of *C. pneumoniae* in the brain of AD patients which may be a risk factor for AD onset and/or pathogenesis. Gérard et al. (2005) studied the burden of *C. pneumoniae* in the AD-brain of patients in respect to Type 4 allele of the apolipoprotein E gene (*APOE-ɛ4)* genotype. They found lower load of *C. pneumoniae*-infected cells in the brain samples from AD patients lacking *APOE-ɛ4*, compared to the regions of AD-brain affected by that allele. It can be concluded that the risk of development AD and its progression to cognitive dysfunction is lower in individuals lacking *ɛ4* allele than individuals bearing this allele.

**Figure 2:**
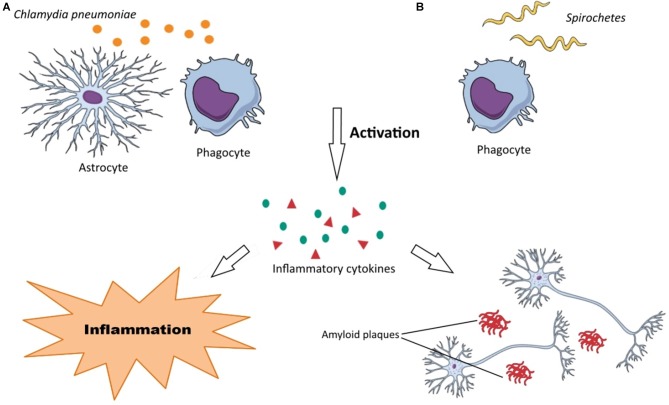
Neuropathogenesis of *Chlamydia pneumonia* and *Spirochetes* species. **(A)** (1) *C. pneumoniae* activates astrocytes and phagocytes (macrophages, microglia, monocytes); (2) the activated forms of these cells produce various inflammatory substances, such as cytokines; and (3) these inflammatory substances render neurodegeneration disorders through neuroinflammation and A*β* accumulation in the brain. **(B)** (1) Recognition of *Spirochetes* species by TLRs on phagocytes (macrophages, microglia) induce their activation; (2) inflammatory substances (chemokines, cytokines, tumor necrosis factor (TNF)) are produced by these cells; and (3) this inflammation develops dementia, cortical atrophy, amyloidosis, A*β* accumulation in the brain and finally cause astrocytosis, microgliosis and neuronal cell loss.

The negative role of *C. pneumoniae* may be attributed to the production of pro-inflammatory cytokines and A*β* accumulation in the brain during chlamydial infections due to chronic inflammation and activation of macrophages and monocytes (Figure 2A). As mentioned earlier, the main mechanism of AD pathology is from A*β* accumulation. Several studies have reported that *C. pneumoniae* principally infects astroglia, macrophages, and microglia in AD patients that are the main cells responsible for inflammation in the brain. The inflammation induced by *C. pneumoniae* plays a significant role in neuroinflammation involved in AD.

The association between *C. pneumoniae* infection and PD has been rarely studied. Turkel et al. (2015) have demonstrated that 98% of PD patients were positive for *C. pneumoniae* IgG, whereas *C. pneumoniae* IgM was negative in both PD subjects and control individuals.

### Spirochetes

Some reports have documented that spirochetes have negative influence on pathogenesis of AD. Historic data indicates that hallmarks of AD are similar to pathological symptoms of general paresis. In one of the oldest study, Noguchi and Moore (1913) discussed the possibility of the presence of *Treponema pallidum* in cerebral cortex of patients suffering from paresis. They have demonstrated that *T. pallidum* can contribute to the development of dementia, cortical atrophy and amyloidosis in atrophic form of general paresis. The pathological hallmarks of this form of paresis include microgliosis, astrocytosis and neuronal cells loss. In addition, the presence plaque-like masses of bacterial colonies in the cortical region of brain was an important pathological evidence of *T. pallidum* involvement in paresis. Moreover, the accumulation of amyloid plaques in the brains of patients with general paresis has been documented.

*Borrelia burgdorferi* is a tick-borne spirochete causing Lyme disease, was also identified in some AD-brains. The first incidence of *B. burgdorferi* in brains of AD cases was reported by MacDonald and Miranda (1987) and the identification was validated using morphological and immunohistochemical features as well as serological methods. Serological methods identified specific antibodies in blood, CSF and neurofibrillary tangles of AD patients. Nevertheless, in these patients neurofibrillary tangles were in co-localized with A*β* and contain specific *B. burgdorferi* genes such as *OspA* and flagellin (Miklossy et al., 2004). In another study (Fallon and Nields, 1994), the involvement of *B. burgdorferi* in neurodegeneration disorders was pointed out through the association of dementia and microgliosis to cortical atrophy in Lyme disease (Fallon and Nields, 1994). Miklossy (2015) examined 147 AD patients for the isolation of *Spirochetes* species by culturing their cerebral cortex and blood on modified Noguchi and Barbor-Stoenner-Kelly II (BSK II) medium. The bacterial isolates were further analyses using scanning electron microscopy and atomic force microscopy for the existence of endoflagella, i.e., the unique characteristic of spirochetes. This study identified spirochetes in blood, cerebral cortex and CSF of 14 AD patients that were absent in control cases. In addition, bacterial peptidoglycan (PGN) was detected using specific antibodies through immunohistochemistry methods. *In situ* hybridization was also applied to identify the species-specific DNA. PGN specific for spirochetes was detected in the brains of 32 AD patients and 12 cases with mild cortical changes (McCoy et al., 2006). Histopathology studies revealed that the spirochetes exist in neurofibrillary tangles, senile plaques and wall of cortical region of AD patients. It is worth mentioning that spirochetes and their specific antigens were also associated with A*β* in the brain suggesting the involvement of spirochetes in dementia and pathogenesis of AD (Miklossy et al., 2004).

There is a large amount of data that reveals spirochetes have a strong neurotropism and can affect the brain cells aggressively causing latent infections. They can also disseminate through lymphatics, nerve fiber tracts, and trigeminal ganglia (Riviere et al., 2002). These microorganisms can be also transmitted via tractus olfactorius. Intriguingly, it has been demonstrated that olfactory tract is affected by spirochetes in AD patients. Spirochetes possess various surface components such as bacterial amyloids, collagen-binding proteins, and pore-forming proteins, which assist them in their attachment onto the surface of host cells (Brissette et al., 2010). Their mechanism of neurodegeneration is probably due to bacterial amyloids causing inflammation and blood coagulation modification through plasminogen and factor XII activation. Host cells, in particular, microglia and phagocytes recognize spirochetes by the receptors that are located on their surfaces. The most important recognition receptors have known to be Toll-like receptors (TLRs), which are also found in the brain (Crack and Bray, 2007). Activated macrophages and microglia produce cytokines and chemokines in response to the spirochetal infections causing inflammation. Following spirochetal infections, A*β* is also accumulated in the brain (Figure 2B). In addition, other specific bacterial components such as D-amino acids and PGN have been also detected in the brain of AD patients. It is noteworthy that the recognition receptors on the surface of host cells have been assumed to be upregulated in the AD-brains. Among them, the activation of TLR2, TLR4 and TLR9 have been reported with remarkable effect on *in vitro* ingestion of A*β* (Minoretti et al., 2006; Tahara et al., 2006). Spirochetes can induce both the classic and alternative immune systems leading to vascular permeability by activating inflammatory responses and clotting cascade. Inflammatory responses may be through spirochetes lipoproteins (i.e., systematic inflammation) or overexpression of macrophage tumor necrosis factor (TNF). Moreover, increasing of serum amyloid A (SAA) and C reactive protein (CRP) levels have been demonstrated in spirochetal infections. Spirochetes have ability to induce systemic inflammation. Interestingly, activated microglia has been observed surrounding the senile plaques in the brain of AD patients (McGeer and McGeer, 2002).

Overall, spirochetes can reproduce the biological, clinical and pathological hallmarks of AD such as A*β* aggregation. More specifically, the lesions in primary neuronal and glial cells as well as brain cell aggregates after their exposures to spirochetes are similar to those occurring in AD (i.e., plaque-like, tangle-like, granulovacuolar degeneration-like lesions). Many studies and reviews have concluded the strong association between AD and spirochetal infection, fulfilling Hill’s nine criteria in the existence of a causal relationship (Miklossy, 2011, 2015).

### Listeria monocytogenes

**Figure 3:**
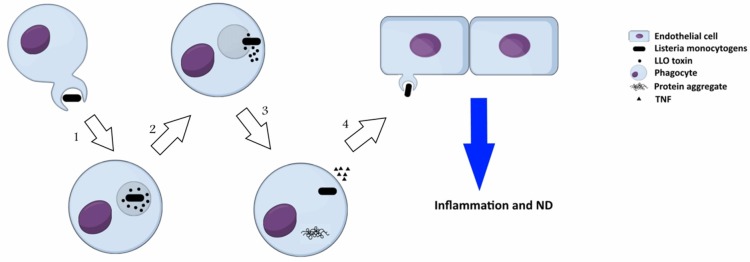
Neuropathogenesis of *Listeria monocytogenes*: (1) secretion of a virulence factor, listeriolysin O (LLO), by *L. monocytogenes* inside phagosome; (2) formation of pre-pore in phagosome, release of the bacterium toxin into cytosol, and onset of invasion in cytosol; (3) formation of LLO aggregates that resembles protein aggregation in neurodegeneration disorders and consists of polyubiquitinated proteins, host protein p62 and LLO, in cytosol of the infected cells; (4) the pathogen spreads from infected phagocytes to endothelial cells and induces neurodegeneration disorders.

*Listeria monocytogenes* is an intracellular, Gram positive bacterium, which is commonly known as a food-born pathogen. *L. monocytogenes* secrets a virulence factor called listeriolysin O (LLO), which contributes in neurodegeneration disorders. LLO is a pore-forming cytolysin that disintegrate the phagosome after internalization of the pathogen into the host cell. This toxin is a member of cholesterol dependent cytolysin (CDC) family that secreted primarily as soluble monomers and oligomerizes to generate a pre-pore at the surface of membrane with high amount of cholesterol. The formation of pores is the consequence of the conversion of two α-helices into *β*-hairpins that extends the membrane further to a bilayer structure to produce a *β-barrel* with a 25 nm channel. It is well-known that protein aggregation is a significant marker in several neurodegeneration disorders such as AD, PD and HD, which can be induced by *Listeria monocytogenes* (Figure 3). Biochemical and molecular methods have indicated the presence of LLO in infected cells. Recently, the association of secreted LLO to the cells with large aggregates has been demonstrated using immunofluorescence approach (Viala et al., 2008). These aggregates are rich in sequestosome1 or p62 and contain polyubiquitinated proteins. Protein p62 is an adapter protein with a PB1 domain in N-terminus accompanied with an ubiquitin domain. N-terminus is responsible for interactions with the subunit S5a/Rpn10 of the proteasome whereas the other domain has the ability to bind polyubiquitin chains. Due to this ability, it seems that p62 behaves as an adapter protein to bind, store and present ubiquitinated proteins to proteasomes. Viala et al. (2008) have studied the LLO aggregates in the cytosol of infected cells with *L. monocytogenes*. They applied specific monoclonal antibodies for LLO detection in marrow-derived macrophages, which were infected with wild-type *L. monocytogenes*. Immunofluorescence approach has revealed the presence of LLO in the cells as both punctuate signal generating circular forms and condensed big groups. LLO aggregates contained polyubiquitinated proteins with host protein p62 and LLO toxin. As aggregation of proteins has been found to be connected with degenerative diseases, LLO accumulation in the cells could resemble those protein aggregation that are associated with neurodegeneration diseases. Furthermore, the infected mono-nucleus phagocytes release TNFα that causes inflammation. This bacterium can even infect endothelial cells directly or spread from infected peripheral blood leukocytes to endothelial cells and cause neuroinvasion.

## Viruses

Retroviruses are a group of viruses containing RNA that cause a wide range of neurodegeneration diseases. Neurovirological diseases are categorized into neurotropism, selective infection of neurons by viruses, and neurovirulence or virus-induced neurological disease. Here, we discuss the viruses that contribute in neurovirulence with focusing on Epstein Bar virus (EBV), herpes simplex virus type 1 (HSV-1), human immunodeficiency virus type 1 (HIV-1), JC virus (JCV), measles virus (MV) and type C or oncogenic viruses. A quick review on neurovirulence of these viruses has been presented in Table 2.

**Table 2:** Neurovirulence of some viruses in human.

Group/Genus	Family	Neurovirulence
Herpesvirus	Epstein–Barr virus	Causing latent and persistent infections in memory B cells to reserve the viral genome. Promoting infectious mononucleosis and triggering an autoimmune response. Enhancing the risk of MS.
Simplexvirus	HSV-1	Causing herpes simplex encephalitis in same sites of brain as in AD patients. Encoding glycoprotein B with high sequence similarity to A*β*. Reducing the production of amyloid precursor protein processing and inducing the production of *β*- and *γ*–secretases for A*β* accumulation.
Lentiviruses	HIV-1	Involving immune system through infection of CD4^+^ T cells and macrophages Crossing the blood-brain barrier with macrophages. Ability to replicate in non-dividing cells such as macrophages or dividing cells such as microglia. Triggering inflammation through the induction of the release of chemokines, cytokines, and excitatory amino acids prostaglandins from infected cells. Causing HIV-associated neurocognitive disorders in different parts of the brain. Reducing the volume of the brain structures and leaving abnormalities in white matter of the brain. Increasing the level of neurofilaments and enhancing the diffusion of A*β* plaques and its subsequent accumulation in the hippocampus and frontal lobe.
*Polyomavirideae*	JC virus	Causing a latent infection in various parts of body such as central nervous system, gut and kidney. Reappearing infections in immunocompromised individuals. Rendering JCV encephalopathy, JCV granule cell neuronopathy, JCV meningitis and progressive multifocal leukoencephalopathy.
*Morbillivirus*	Measles virus	Infecting lymphocytic cells, then replicating in lymph nodes. Triggering a strong leucopenia. Crossing the blood-brain barrier with leukocytes or infecting microvascular endothelial cells to invade brain via migration from choroid plexus or olfactory bulb. Inducing acute post infectious measles encephalitis or subacute sclerosing panencephalitis after measles period.
Oncogenic viruses/type C	HTLV-1	Malignancy or neurological syndrome in human.
	HTLV-2	HTVL-1 causes adult T-cell leukemia/lymphoma. Inflammatory disorders such as progressive myelopathy or HTLV-1-associated myelopathy.
	MuLV	Infecting astrocytes, brain endothelial cells, leucocytes, microglia, and oligodendrocytes. Causing spongiform changes in the spinal cord and brainstem as well as behavioral deficiencies such as hind limb paralysis and tremor. Activating glial cells and increasing the release of pro-inflammatory substances. Promoting the neuronal cell death.

### Epstein–Barr Virus

EBV infections predominantly occur in childhood with no apparent symptoms. However, it can emerge with clinical symptoms such as infectious mononucleosis, particularly, in adults. It has been estimated that approximately 90% of people are infected with EBV worldwide. It is generally accepted that EBV is associated with some autoimmune diseases such as human tumors and MS (up to 99%). This consistency may be explained by the ability of this virus to cause latent and persistent infections in memory B cells to reserve the viral genome. Interestingly, infectious mononucleosis induced by EBV can remarkably increase the risk of MS as individuals with anti-EBV nuclearantigen-1 (EBNA-1) IgG and anti-VCA IgG antibodies have an enhanced MS risk (Santiago et al., 2010). Increasing the anti-EBNA-1 antibodies results in cross reactivity with self-antibodies in MS patients. It has been indicated that EBNA-1 has cross reactivity with heterogeneous nuclear ribonucleoprotein L (HNRNPL), introducing the HNRNPL as an autoantigen (Lindsey et al., 2016). Cross-reactivity of monoclonal myelin basic protein (MBP)-specific antibodies obtained from MS patients with latent membrane protein 1 (LMP1) of EBV is another example of autoimmune response in MS. More specifically, the injection of LMP1 to mice causes myelin-reactive autoantibodies induction (Lomakin et al., 2017). As mentioned earlier, most of the adult individuals infected with EBV do not show MS symptoms, suggesting EBV alone is not adequate for development of MS. However, its combination with other factors including EBV variant, vitamin D deficiency and genetic susceptibility of immune system such as MHC class II gene can cause MS (Najafipoor et al., 2015).

There is a controversy about the presence of EBV in the brain of MS patients and its association with MS pathogenesis. Some studies have mentioned that this virus is residing in B cells of the brain and in meningeal follicle-like structures whereas other reports have expressed that there are no signs of EBV or there is only small population of virus-infected B cells in the brain of MS patients. Torkildsen et al. (2010) examined cortical gray matter lesions of MS patients and observed upregulation of immunoglobulin-related genes in these sections by plasma cells, rather than accumulation of B cells. Moreover, they could not detect any evidence of active or latent EBV infection. This finding maybe due to the fact that gray matter lesions are pathologically different from white matter lesions showing absence or paucity of infiltrating immune cells including lymphocytes. In another study, (Willis et al., 2009) used real-time PCR for the detection of viral genome and the amplification of EBV-encoded RNA from white matter lesions of 17 MS patients. No positive results were observed except a signal indicating the expression of CD20 messenger RNA and the presence of CD20^+^ B cells. In addition, another set of samples including B cell aggregates in parenchyma and free B cell infiltrate in the meninges examined and showed minute amounts of EBV RNA only in two of 12 samples MS patients. In contrast, the detection of EBV-encoded small RNA (EBER) using* in situ* hybridization approach confirmed the presence of EBV in the brain of MS patients (Serafini et al., 2007). EBER1 and EBER2 are two untranslated transcripts of EBV that are principally located in the cell nucleus and expressed in latent infection of the virus. Using this method, high number of EBER-positive cells from white matter and meninges of MS subjects have been found that were mostly in B cell follicles (Serafini et al., 2007).

Overall, the direct contribution of EBV infection in MS immunopathology is unlikely; however, it may contribute to MS through indirectly influencing immune function or via molecular mimicry between CNS and EBV antigens (Willis et al., 2009).

### Herpes Simplex Virus Type 1

HSV-1 infection usually creates a skin infection termed as cold sores, which occurs in the early age of individuals and starts replicating in the initial infected place (mostly in lips). Thereafter, the virus moves to nerve cells and enters into latency phase in peripheral nervous system. In this stage, the genome of virus can be found but viral proteins are undetectable. When the virus leaves the latency phase, it begins to replicate and to produce viral proteins and whole virions, leading to acute infection. HSV-1 causes herpes simplex encephalitis (HSE) in the same sites of the brain as in AD patients. Additionally, HSV-1 may involve in AD due to the virus ability for being long-time latent in nerve cells as well as the presence of active form of virus in large number of elderly people. Similar to AD, the risk of cold sores is higher in the individuals carrying* APOE-ɛ4* (Wozniak et al., 2009).

HSV-1 can encode glycoprotein B with high sequence similarity and characteristics to A*β* and reduces amyloid precursor protein (APP) processing. More specifically, the neuronal cells infected by HSV-1 displayed a lower level of APP, a large increase in 55 kDa APP fragment, and higher intracellular levels of A*β*. Additionally, HSV-1 induced the production of enzymes responsible for A*β* formation, *β*- and *γ-secretases*, which are accumulating A*β* in HSV-1-infected cells and mice brain leading to amyloid plaques (Wozniak et al., 2009). Accordingly, this pathogenesis of HSV-1 introduce this virus as an important potential etiological factor in AD.

### Human Immunodeficiency Virus Type 1

Lentiviruses consist of important members, including various immunodeficiency viruses of bovine (BIV), feline (FIV), human (HIV), simian (SIV) and visna-maedi virus (VMV). Although Lentiviruses encompass diverse lentiviral genes, they contain common genetic and biological characteristics such as retroviral genome organization that are essential for their virulence. They have the ability for replication in non-dividing cells such as macrophages. In addition, lentiviruses often prefer to infect macrophages and microglia rather than astrocytes and endothelial cells. The neuropathogenic mechanism in lentiviruses is assumed to be through the infection of macrophages entering CNS by crossing the blood-brain barrier. Cell-surface receptors for some lentiviruses (e.g., HIV and SIV) have been detected inside and outside of nervous system such as CD4, chemokine receptors and CC chemokine receptor 5 (CCR5; Power, 2001). Neuropathology of lentiviruses is determined by several factors such as host age and its immune system status. It also depends on individual lentiviruse strain. Lentiviruse-infected patients develop dementia or encephalopathy accompanied by inflammation and neuronal cells injury. The basal ganglia and frontal cortex have been known as the main sites for macrophages infiltration and infection, rendering neuronal cells of these parts vulnerable to lentiviruses infections. Lentivirus induced infections severely modify neuronal cells and caused endritic vacuolization, axonal damage, neuronal loss accompanied by changes in blood-brain barrier permeability and its function (Everall et al., 1999).

The infection of brain cells with HIV-1 can lead to neuroinflammation, motor dysfunction and syndrome HIV dementia. HIV-1 principally infects macrophages and microglia leading to the release of chemokines, cytokines and excitatory amino acids prostaglandins from infected cells. These released factors trigger inflammation and neuronal injury. Based on HIV tropism to the human cells, HIV-1 is categorized into M- and T-tropic strains. Macrophages are targeted by M-tropic HIV-1 strains through CC chemokine receptor 5 (CCR5), whereas T-tropic strains use G protein-coupled CXC receptor 4 (CXCR4) for infecting macrophages. Chemokine SDF-1 is a significant molecule that can bind CXCR4 making this receptor unavailable for HIV-1 binding. It also has an important role in the migration of leukocytes and induction of the intracellular signaling in leukocytes (Albright and González-Scarano, 2004).

The virus has capability to predominately involve immune system with infection of CD4^+^ T cells and macrophages. Prior to finding of an effective treatment, 8%–15% of patients with HIV infection suffered from a severe cognitive disease, called HIV-associated dementia (HAD). This number has been reduced to 2% after the development of combination antiretroviral therapy (cART; Heaton et al., 2010). However, using cART dose not completely eliminate the virus from the brain cells, so the patients with HIV may have chronic infection in CNS. It is noteworthy that most antiviral drugs cannot effectively penetrate into the brain; therefore, viral infection keeps progressing in the CNS. HIV-associated neurocognitive disorders (HAND) referred to neurocognitive impairments including AIDS dementia complex, HIV encephalitis, HIV encephalopathy and minor cognitive motor disorder showing various clinical features. Pathophysiology of HAND includes inflammation and neuronal cells dysfunction. Imaging studies and CSF examination have revealed that inflammation occurs in HIV infection within 18 days after the virus exposure (Valcour et al., 2012) through activation of macrophages leading to CNS disorder. HIV infected macrophages activate microglia and results in emerging giant cells with multi-nucleus and astrogliosis diffusion. Consequently, this response contributes in HIV encephalopathy, neuronal cells loss and neurodegeneration in different parts of the brain. Magnetic resonance spectroscopy has detected some changes in metabolite quantity of the brains of HIV patients. This effect is associated with inflammation, gliosis and neuronal cells loss and has been even observed in cART-treated individuals (Harezlak et al., 2011). Magnetic resonance imaging showed that the volume of brain structures has shrunk in HAD individuals. In addition, Diffusion Tensor Imaging, a useful technology to measure the water molecules diffusion, has revealed abnormalities in white matter of HAD brain of patients. cART treatment may improve the abnormalities but it does not completely restore the normal status. Furthermore, the measurement of metabolic activity in the brain using Positron Emission Tomography technology has demonstrated regions in HAD brain with altered metabolism that are correlated with cognitive status (von Giesen et al., 2000).

Neurofilaments (NFs), i.e., specific neuronal proteins with low, medium and high molecular weight may be found in blood stream and CSF as a result of neurons loss or disruption. This is considered as a significant biomarker in neurodegeneration disorders such as AD, ASL, MS and subcortical vascular dementia (Malmeström et al., 2003; Pasol et al., 2010). Notably, the levels of NFs in CSF of HIV patients increase and are significantly higher in HAD individuals. It has been demonstrated that cART treatment can decrease the NFs levels, followed by improvement of cognitive impairment (Jessen Krut et al., 2014). In addition, neuronal damage is correlated with macrophages and monocyte activation; and neopterin, a biomarker of macrophages and monocytes activation, has been detected in CSF of HAD patients. Membrane-bound CD14, a receptor for lipopolysaccharide, is a marker of monocytes activation that is cleaved and converted into soluble form, i.e., sCD14. Measurement of plasma sCD14 levels has revealed the correlation between this biomarker and impaired neurocognitive in HIV patients (Lyons et al., 2011). Moreover, another macrophages/monocytes activation marker, CD163, increases in cognitive impairment HIV patients (Burdo et al., 2013).

The hallmark of AD, i.e., A*β* accumulation also occurs in HIV patients. In HIV patients A*β* plaques diffuse and subsequently accumulate in the hippocampus and frontal lobe. Pathological studies showed that the accumulation of A*β* in AD brain of patients commonly occurs in neocortical regions.

Although, many data highlights the strong correlation between A*β* accumulation in the brain and AD, only a few number of studies suggest this abnormality as a main contributor in HAND pathology (Cohen et al., 2015). HIV can induce the permeability of blood-brain barrier through disruption of microvascular endothelial cells integrity and tight cell junctions, which facilitates the access of infected macrophages to the brain. Blood-brain barrier integrity disruption affects the A*β* accumulation in the brain of HIV patients due to its disability to filter the A*β* peptide suggesting the similar neuropathological features of AD and HIV (Erickson and Banks, 2013).

### JC Virus

JCV is a neurotropic circular double stranded DNA virus belonging to *Polyomavirideae*. The virus shares up to 70% genomic similarity with BK virus and SV40. Its genome comprises three main parts including an early coding region, a late coding region, and a regulatory region. The virus usually causes a latent infection in various parts of body such as CNS, gut and kidney. Reappearing of JCV infections including JCV encephalopathy (JCVE), JCV granule cell neuronopathy (GCN), JCV meningitis, and progressive multifocal leukoencephalopathy (PML) often occurs in immunocompromised individuals with different types of diseases.

PML is a dangerous JC virus-induced demyelinating disease in patients with impaired immunity. The hallmark of the disease is infection of oligodendrocytes with the presence of enlarged nuclei and intracellular inclusions. The infected cells are usually observed in peripheral parts of well-demarcated demyelinating lesions or disturbed regions in myelin pallor cases. Neuropathology of PML is associated with diverse clinical symptoms such as demyelinating lesions in the cerebral subcortical white matter and cortical region (Moll et al., 2008). The most affected region is probably frontal lobes and parietooccipital parts, and, to a lesser extent, in cerebellum, the brainstem and spinal cord of PML patients (Bernal-Cano et al., 2007). Intensity of demyelination of cells varies from myelin pallor to demyelination accompanied with axonal damage and macrophages carrying myelin debris generating burnt out lesions that often are seen in AIDS patients (Gray et al., 2003).

GCN is another JCV-associated disease characterized by emerging lytic infection of granule cell neurons resulting in loss of neurons and cerebellar atrophy. Most cases with GCN have been reported in HIV patients (Agnihotri et al., 2014). The neurodegeneration has been observed in some patients suffering from GCN by imaging findings. White matter modifications, especially, in middle cerebellar peduncles and the pons have been observed in association with gray matter in these patients. Additionally, it has been documented that GCN can occurs in astrocytes and oligodendrocytes (Wijburg et al., 2015).

JCVE has been introduced as a new neurodegenerative syndrome caused by JCV in 2009. This disease is characterized by detecting the lesions restricted to gray matter and the presence of virus in cortical gray matter as well as CSF of patients. JCVE predominantly affects cerebral pyramidal neurons and astrocytes in hemispheric gray matter. The presence of viral macromolecules such as viral proteins in axons neurons have been reported suggesting the migration of JCV to the brain through infection of axons of neurons (Miskin and Koralnik, 2015).

### Measles Virus

MV, a member of genus *Morbillivirus*, is known as a highly cell associated virus. The virus enters the host body through respiratory tract and moves to lymphocytic cells, then replicates in lymph nodes. In early stages of the infection, it can be easily isolated from blood leukocytes. A strong leucopenia occurs in patients during measles due to an increase in the attachment of lymphocytes to the lymphoid organs. After replication of the virus in lymphoid organs, it can be found in other tissues and cells such as skin, endothelial cells and brain. Access to the brain cells occurs through various ways including transfer of infected leukocytes through blood-brain barrier, infection of microvascular endothelial cells and migration via choroid plexus or olfactory bulb (McQuaid and Cosby, 2002). The increase in leukocytes adhesion during MV infection reduced the migration capacity of the cells across the endothelial barriers and enhanced the infection of endothelial cells (Dittmar et al., 2008).

After measles period, MV can induce two different forms of infection in CNS: acute post-infectious measles encephalitis (APME) or subacute sclerosing panencephalitis (SSPE). It has been estimated that AMPE, the most prevalent CNS complication, observes in 0.1% of MV-infected patients with a mortality rate of 20%. According to the electroencephalogram data, 50% of patients suffer from cerebral dysfunction. The presence of MV nucleic acid has been detected only in CNS of APME patients. Therefore, the clinical symptoms may be related to autoimmune response of the host. Inflammatory response may be initiated by infected endothelial cells of the brain or by attached leukocytes to brain microvascular endothelial cells (Reuter and Schneider-Schaulies, 2010). In contrast, SSPE is a rare, progressive neurodegeneration disease that occurs after years of viral persistence. MV variant isolated from the CNS of these patients differs from wild-type virus. The former is deficit and its genome structure harbors various mutations that caused primarily through the replacement of uridine by cytidine in the matrix gene (M gene). *In vitro* and *in vivo* studies have revealed that despite biased hyper-mutations in M gene, the mutant virus has capability to infect the brain of adult mice and cultures of primary neurons (Patterson et al., 2001). In general, the reason for persistence of MV in the CNS remains unexplained and the paucity of SSPE patients has further complicated this line of investigation. On the other hand, in human neurons, the known receptors for MV (i.e., nectin 4 and CD 150) are not expressed and the mechanism of MV propagation in these cells is still unclear. The spread of the MV variant in neurons essentially requires destabilizing hyperfusogenic modifications in the F protein ectodomain, allowing the virus to display tropism for neurons (Watanabe et al., 2018).

### Oncogenic Viruses or Type C

Oncogenic viruses are a group of retroviruses that causes neurological diseases in human and some animals such as bird, cat and rodents. The genome of these viruses comprises three main structural genes, including two LRT parts and an accessory gene. Avian leukemia viruses (ALV), human T-cell lymphotropic virus types 1 and 2 (HTLV-1 and HTLV-2) and murine leukemia viruses (MuLV) are the major groups of neurotropic oncogenic retroviruses.

#### HTLV-1 and HTLV-2

HTLVs are persistence viruses that can efficiently transform T lymphocytes and immortalize them. Compared to HTLV-1, both CD4^+^ and CD8^+^ T cells have similar susceptibility towards HTLV-2; however, the latter virus is much less frequent and pathogenic. More specifically, HTLV-1 preferentially exhibits transformation tropism for CD4^+^ T cells, whereas HTLV-2 shows higher proviral burden in CD8^+^ T cells. Malignancy or neurological syndrome (incidence rate <1%) is an example of neurological disorders caused by HTLV viruses in human. HTLV-1 and HTLV-2 induce a progressive myelopathy defined by inflammation in the thoracic part of the spinal cord (Power, 2001). It has been estimated that HTLVs afflict about 20 million individuals worldwide. The infected individuals may be either asymptomatic or neurological; both of them can potentially transmit the virus.

HTLV-1 causes a lymphoproliferative malignancy that is called adult T-cell leukemia/lymphoma. Moreover, the virus can induce inflammatory disorders such as HTLV-1-associated myelopathy (HAM/TSP) in children and female individuals. Initial findings on the pathology of HAM/TSP showed the effect of HTLV-1 Tax-specific CD4^+^ cytotoxic T lymphocytes. A local parenchymal damage is possible by releasing cytokines and lymphokines during the process of T-cell transforming. Host autoimmune response is another important parameter triggered during the pathogenesis of HTLV-1 in HAM/TSP patients. These patients produce IgG antibodies that cross-react with both HNRNP–A1 and an immunodominant epitope in Tax (Levin et al., 2002).

The first report of infection caused by HTLV-2 was related to the patients with both HTLV-1 and HTLV-2 infections and myelopathy. It has been revealed that there are some patients with symptoms of HAM/TSP with single HTLV-2 infection, suggesting the direct role of HTLV-2 in HAM/TSP disorder.

#### Murine Leukaemia Viruses

MuLVs are known as the largest group of neurotropic retroviruses that causes some behavioral deficiencies such as hindlimb paralysis and tremor. These types of retroviruses often infect astrocytes, microglia and oligodendrocytes. However, there are some MuLV strains, which infect neuronal cells. MuLVs strains employ various cell-surface receptors to attach the cells such as mCAT-1, although there is no evidence for the role of this receptor in nervous system. The infection of leucocytes and brain endothelial cells by MuLVs are the most probable manner to access the brain. Although the type of neuropathology caused by these viruses depends on strain, spongiform changes in the spinal cord and brainstem have been known as the most common causes of pathology. The mechanisms by which MuLV causes infections in hosts often associated with interaction between sequences in virus genome, particularly located in *gag* or *env* genes, with host responses. Two domains in the *env* gene of Fr-98 MuLV control the neurovirulence in mouse model and cause abnormal behavior and death (Poulsen et al., 1998). Although there is little evidence for pathogenic host responses in MuLV infection, the activation of glial cells and increased pro-inflammatory production is considered as the main pathways of neurovirulence. In addition, MuLV infections mediate a surge in glutamate concentrations in extracellular environment through alteration in permeability of blood-brain barrier, leading to neuronal cells death (Sei et al., 1998).

## Fungi and Yeasts

### Footprint of Fungi in AD Patients

Recently, the role of fungi in neurodegeneration diseases has been considered by a number of studies. Accordingly, fungal proteins, DNA and polysaccharides have been found in blood and CSF of AD patients. Additionally, fungal cells have been directly detected in different sections of CNS in AD individuals including frontal cortex, cerebellar hemisphere, entorhinal cortex/hippocampus and choroid plexus using immunohistochemistry and confocal microscopy. In addition, fungal DNA, proteins and polysaccharide were observed in blood serum from AD patients. The fungal species present in CNS samples from AD patients belonged to *Cladosporium*, *Malassezia, Neosartorya, Phoma, Saccharomyces, Sclerotinia*, and* Candida* species (Pisa et al., 2015). Moreover, Alonso et al. (2014) have identified the fungal DNA and proteins in CSF using PCR and slot-blot assay, respectively.

### Fungal Infections in MS Patients

The autoimmunity in MS is thought to be triggered by exotic proteins or antigens that mimic self-molecules leading to reaction against myelin followed by destruction of glial and neuronal cells. The presence of fungal macromolecules such as proteins and DNA in CSF of MS patients have been studied by Pisa et al. (2015). They have shown that fungal proteins can be detected in CSF of several MS patients while fungal DNA has been amplified in some samples. PCR results have demonstrated the presence of different fungal species in the CSF samples of MS patients.

There is evidence which links some yeasts infections with MS. Genus *Candida* has been well-recognized as a pathogenic yeast for human, rendering demyelinated lesions in CNS of patients infected with *Candida* species (Lipton et al., 1984). As the *Candida* species are found as commensal organisms in several parts of human body, such as GI tract, oral cavity, and skin, antibodies against these species are found in normal individuals. However, indirect immunofluorescence approach has revealed the significant antibodies titter against *Candida* species in MS patients (Benito-León et al., 2010). It has been hypothesized that clinically pathogenic fungi and yeasts such as species of *Aspergillus* and *Candida* have ability to mask themselves from immune system by applying their mannoprotein coat, which help them attach to the non-neuronal tissues and release their toxins (mycotoxins) to the bloodstream (Purzycki and Shain, 2010). Mycotoxins pass through the blood-barrier brain and affect astrocytes and oligodendrocytes, which their significant roles are maintenance of the blood-brain barrier integration and nutritive support for the myelin. Consequently, these toxins renders myelin susceptible to degradation by various factors, which is the main hallmark of MS (Purzycki and Shain, 2010). *Fusarium* species produce a kind of mycotoxins called Fumonisin B, which interferes in sphingolipids biosynthesis. The loss of sphingolipids in white matter is a characteristic symptom of MS patients (Stockmann-Juvala and Savolainen, 2008). In addition, Fumonisin B has toxicity effect on primary astrocytes and murine microglia (Stockmann-Juvala and Savolainen, 2008). Penitrem A is produced by *Penicillium crustosum*, which can involve in various neurological disorders such as ataxia, mitochondrial swelling, nystagmus and pseudoparalysis in animal models (Cavanagh et al., 1998).

Gliotoxin has been isolated from species of *Aspergillus* and *Candida*, which causes various mycotoxicoses (Reeves et al., 2004).This heat stable toxin uses glutathione-dependent redox pathway to keep its intracellular concentrations >1,000 times of its extracellular toxin levels. *In vitro* and *in vivo* studies revealed that gliotoxin targets CNS astrocytes in rat models (Willis et al., 2004). Moreover, heat-treated CSF obtained from MS patients triggers apoptotic death of astrocytes and oligodendrocytes (Purzycki and Shain, 2010). It is noteworthy that CSF from patients with other inflammatory or non-inflammatory neurological disorders showed no toxicity against these cells. Other study has implicated that intraventricular injection of CSF containing gliotoxin increases permeability of blood-brain barrier leading to immunoglobulins leakage (Rieger et al., 1996).

## Conclusion

For many decades, the causative agents leading to neurodegeneration disorders were unknown. It is generally accepted that many neurodegeneration disorders are the results of genetic mutations, inflammation, misfolded proteins and environmental factors. While this holds true, it does not consider the contribution of other diseases. Another assumption is the association of microbial infections and etiology of neurodegeneration diseases. This assumption even reasonably provides direct relation with age, as individuals with older ages exposed to a higher number of microbial diseases and also have higher risk to reveal neurodegeneration disorders. Microorganisms can affect brain development, levels of neurotransmitters, levels of brain-derived neutrophilic factor and levels of amygdale. Upon infection, microorganisms can induce the release of various cell signaling proteins that cause inflammation. When these microorganism pass blood-brain barrier, this inflammation commonly ends with irreversible neuron degenerations and produces similar hallmarks to various neurodegeneration disorders. The impact of microbial diseases treatment on neurodegeneration diseases is an interesting area for future work and it may at least postpone the onset of these diseases. Furthermore, the potential incidence of neurodegenerative disorders in any given population may be determined through immune system vulnerability to neurodegeneration diseases associated microbial diseases. Additionally, the health history of any individual with neurodegeneration diseases associated microbial diseases may explain their susceptibility to neurodegeneration disorders. A further understanding of the basis for these relations may contribute in the generation of novel remedies or some sort of immunity for neurodegeneration disorders and should remain a focus of intense cases and molecular studies.

## Author Contributions

MD and HKSP both wrote the manuscript and GG edited, approved and finalized it.

## Conflict of Interest Statement

The authors declare that the research was conducted in the absence of any commercial or financial relationships that could be construed as a potential conflict of interest.
